# Colorectal cancer in patients seen at the teaching hospitals of Guadeloupe and Martinique: discrepancies, similarities in clinicopathological features, and p53 status

**DOI:** 10.1186/1472-6890-14-12

**Published:** 2014-03-29

**Authors:** Monique Decastel, Marlene Ossondo, Anne-Marie Andrea, Benoît Tressieres, Jacqueline Veronique-baudin, Jacqueline Deloumeaux, Marc Lubeth, Juliette Smith-ravin

**Affiliations:** 1 UMR Inserm_S1134, Université des Antilles et de la Guyane (UAG), CNRS SNC 9169, Pointe-à-Pitre, Guadeloupe; 2 Department of Anatomopathology, Teaching Hospital of Zobda Quitman, Fort de France, Martinique, France; 3 Department of Anatomopathology, Teaching Hospital of Pointe-à-Pitre, Pointe-à-Pitre, Guadeloupe; 4 Centre d’Investigation Clinique-EC Antilles Guyane (CIE 802 Inserm), Teaching Hospital of Pointe-à-Pitre, Pointe-à-Pitre, Guadeloupe; 5 Cancer Registry of Martinique (AMREC), Fort de France, Martinique; 6 Cancer Registry of Guadeloupe, Teaching Hospital of Pointe-à-Pitre, Pointe-à-Pitre, Guadeloupe; 7 Department of Digestive Surgery, Teaching Hospital of Pointe-à-Pitre, Pointe-à-Pitre, Guadeloupe; 8 UAG, Département Scientifique Interfacultaire, EA929 AIHP-GEODE BIOSPHERES, Campus de Schœlcher, Martinique 97200, France; 9 UMR_S1134 Inserm, CHU de Pointe-à-Pitre, bâtiment Ricou, 97159 Guadeloupe, France

**Keywords:** Colorectal cancer, Guadeloupian patients, Martinican patients, Clinicopathology, p53 status, Discrepancy

## Abstract

**Background:**

In Guadeloupe and Martinique, two French Overseas Departments, colorectal cancer (CRC) has become an essential public health issue. However, little is known about CRC characteristics and the p53 status in these populations, particularly in Guadeloupe, whereas certification of a cancer registry has been recently validated.

**Methods:**

This was a descriptive retrospective study of 201 patients who, between 1995 and 2000, underwent surgery for CRC in the Guadeloupe Teaching Hospital (GlpeTH; 83 patients) and in the Martinique Teaching Hospital (MqueTH; 118 patients). The clinicopathological features and the p53 expression, evaluated with immunohistochemistry, were compared at the time of diagnosis. A relationship between these parameters and the p53 expression was also studied. Data were analysed, using the SPSS computer software version 17.0.

**Results:**

No statistical difference was found between the two groups of patients regarding age (p = 0.60), percentage of young patients (≤50 years; p = 0.94)), sex (p = 0.47), histological type (p = 0.073) and tumour sites (p = 0.65), although the GlpeTH patients were diagnosed with more distal colon cancers (54.2%) than the Mque TH patients (47.4%). By contrast, a significant difference was found regarding the tumour grade (p < 0.0001), the pTNM stage (p = 0.045) and the pT stage (p < 0.0001). Regarding p53 expression, solely for the MqueTH patients, nuclear expression was associated with pTNM, the percentage of p53 negative tumours increasing with the progression of the pTNM stages (p = 0.029).

**Conclusions:**

For the first time, this study reveals discrepancies in clinicopathological features and in the p53 status between the two groups of patients. The GlpeTH patients were diagnosed with more moderated CRCs but with few CRCs at pTNM IV stage. By contrast, the MqueTH patients were diagnosed with more differentiated tumours, but with many more CRCs at pTNM IV stage. This paradox may be due to differences in tumour location (distal vs proximal), multiplicity of the genetic profiles of patients, or patients getting treatment elsewhere. Although our study is limited due to its small size, it emphasizes the originality of our results.

## Background

Colorectal cancer is a major cause of morbidity and mortality in men and women worldwide [1,2]. Incidence in France is comparable to the one found in other high-risk areas like Western Europe, North America, Australia/New Zealand and Japan [2], Africa and Asia being lower-risk areas [1]. Despite considerable efforts made for the last decades, CRC remains one of the most frequent, deadly cancers triggered by environmental and genetic factors [3]. CRC develops through a series of progressive changes defined by clinical and histopathological parameters [3,4]. Genetic changes, including inactivation of the tumour suppressor gene p53, have been often associated with the different steps of neoplastic progression in CRC [5-7]. The p53-suppressor gene is the most frequently mutated gene identified in solid human malignancy [6]. Mutation of the p53 gene results in the production of a protein with altered growth regulatory properties and with a conformational change [7-9]. The latter feature prolongs its lifespan, stability and accumulation, enabling p53 detection with routine immunohistochemistry (IHC) [9,10].

Colorectal cancer in Guadeloupe and Martinique, two French overseas regions in the Caribbean, with a similar health care system, a similar medical practice as well as a similar socio-economic status, is the second most common cancer in men and the third in women, becoming an important public health problem. The incidence of CRC has increased five-fold in 25 years. The main factors for this increase are not known, but may be due to changes in dietary habits, and/or to environmental exposures. For example, in 1995, twenty-five and thirty-seven cases of CRC were diagnosed in the GlpeTH and in the MqueTH, respectively. In 2008, 138 cases were diagnosed in Guadeloupe, versus 189 cases in Martinique [11]. Nevertheless, there are little data regarding the patterns of CRC, particularly in Guadeloupe. As far as we know, there are only three studies which have been published on this topic: one of Serra et al. [12] on the comparison of cancer morbidity between Guadeloupe, Martinique and French metropolitan regions; one of Ngasseu et al. [13] on CRC incidence and mortality rates in Martinique, and, finally, the study of Dieye et al. [14] on epidemiological cancer transition in Martinique.

The purpose of this retrospective study was to describe the clinicopathological characteristics of CRC in patients who, between 1995 and 2000, underwent surgery in the GpleTH and in the MqueTH. In addition, the p53 protein expression being not known, we also studied its pattern of expression and we determined its association with the clinicopathological parameters analysed.

## Methods

### Patient information and tissue samples

This descriptive retrospective study was approved by the local Ethical committee of both teaching hospitals. In the GlpeTH, 121 patients were seen for CRC between 1995 and 2000. Twelve patients (9.9%) were lost to follow up and 109 patients underwent surgery. Out of the 109 patients, 24 who only had biopsies were excluded, as well as 2 who did not have complete clinical data. Finally, 83 patients were eligible. In the MqueTH, a total of 118 patients were eligible, 4 being excluded for absence of clinical data. Archival formalin-fixed and paraffin-embedded tumour tissues and normal mucosa, taken 5 cm away from the tumour area, were available for a total of 201 CRCs. None of the patient had received chemotherapy or radiotherapy prior surgical resection. Two pathologists (MO, AM) individually reviewed all the haematoxylin/eosin stained slides. Histological type, tumour grade and tumour stage were determined according to the WHO and TNM classification systems. Regarding cancer location, the cases were grouped either into 4 categories: right colon, left colon, sigmoid colon and rectum, or three categories: proximal colon (right and transverse), distal colon (left and sigmoid) and rectum, when appropriate [4].

There are at least three reasons why we selected the 1995–2000 period: Firstly, in 1995, one could notice that the percentage of black people descending from deportations of Africans as slaves has evolved, leading to disparities between Martinique and Guadeloupe [15]. Indeed, the percentage of mixed raced population was greater in the former (91%) than in the latter (86%); inversely, the percentage of black people was lower in Martinique (6.2%) than in Guadeloupe (11.6%). Secondly, from 1995 on, a significant increase in CRC incidence, strengthened by the clinicians of the Department of Medical information of the GlpeTH, could be observed, whereas the French National Commission of Data Processing and Civil Liberty (CNIL) has recently authorized the establishment of a cancer registry for Guadeloupe. Thirdly, data from the MqueTH patients were available for the same period of time (1995–2000), which makes the comparison between the GlpeTH and MqueTH patients easier, as the two French overseas regions have a similar health care system and similar medical practices.

### Immunohistochemical detection of p53 and staining evaluation

For immunohistochemistry (IHC) all the slides were treated in the department of anatomopathology of MqueTH to avoid discrepancy. Four-μm sections obtained from the archival paraffin embedded tissues (tumour and normal adjacent) were deparaffinised using toluene and a graded series of ethanol. After rehydration, endogenous peroxidase activity was blocked with 3% hydrogen peroxide in absolute methanol and then, microwave antigen retrieval was performed in a 10 mM citrate buffer for 30 minutes at high power before antibody labelling. The immunohistochemical procedure was performed with a Ventana auto-immunohistochemical stainer (Illkirch, France) according to the manufacturer’s guidelines. The mouse monoclonal antibody (clone DO-1; IgG2a; Immunotech, Marseille, France), which recognises both wild-type and mutant forms of human p53 protein, was used at a dilution of 1:50. Primary antibody was omitted in the negative control. The antigen-antibody complex was visualized using the Ventana/View detection kit for biotin streptavidin-horseradish-peroxydase. Slides were counterstained with haematoxylin before mounting. The positive reaction, shown by a brown colour, was evaluated under a light microscope both at a low and a high power and was scored by two pathologists (MO, AM) who were blinded to the origin of the sections and to the clinicopathological features of the patients. Compared with the negative control and/or the adjacent normal mucosa, p53 staining was considered as positive when the tumour cell nuclei were stained, irrespective of the percentage of positive cells; the staining was scored on semi-quantitative scales as follows: no reaction (0), weak reaction (+), moderate reaction (++) and strong reaction (+++). In the cases where there was initial disagreement, a consensus was obtained after discussion. Finally, 76 slides from the GlpeTH and 117 slides from the MqueTH, were analysed.

### Statistical analyses

Statistical analyses were performed using the Statistical Package for the Social Science (SPSS) computer software version 17.0 (IBM SPSS Statistics, Chicago, IL, USA). The Chi-square test or Fisher’s exact test was used when appropriate, for comparing categorical variables (contingency tables). The Student t test was used to compare differences between quantitative variables. Various typical prognostic factors were considered for univariate analysis. Multivariate analysis (logistic regression) was performed for adjusting p53 group overexpression (negative p53 versus positive p53) with clinicopathological parameters. A p value less than 0.05 was considered statistically significant.

## Results

### Description of patients according to clinical and pathological features

**Figure 1:**
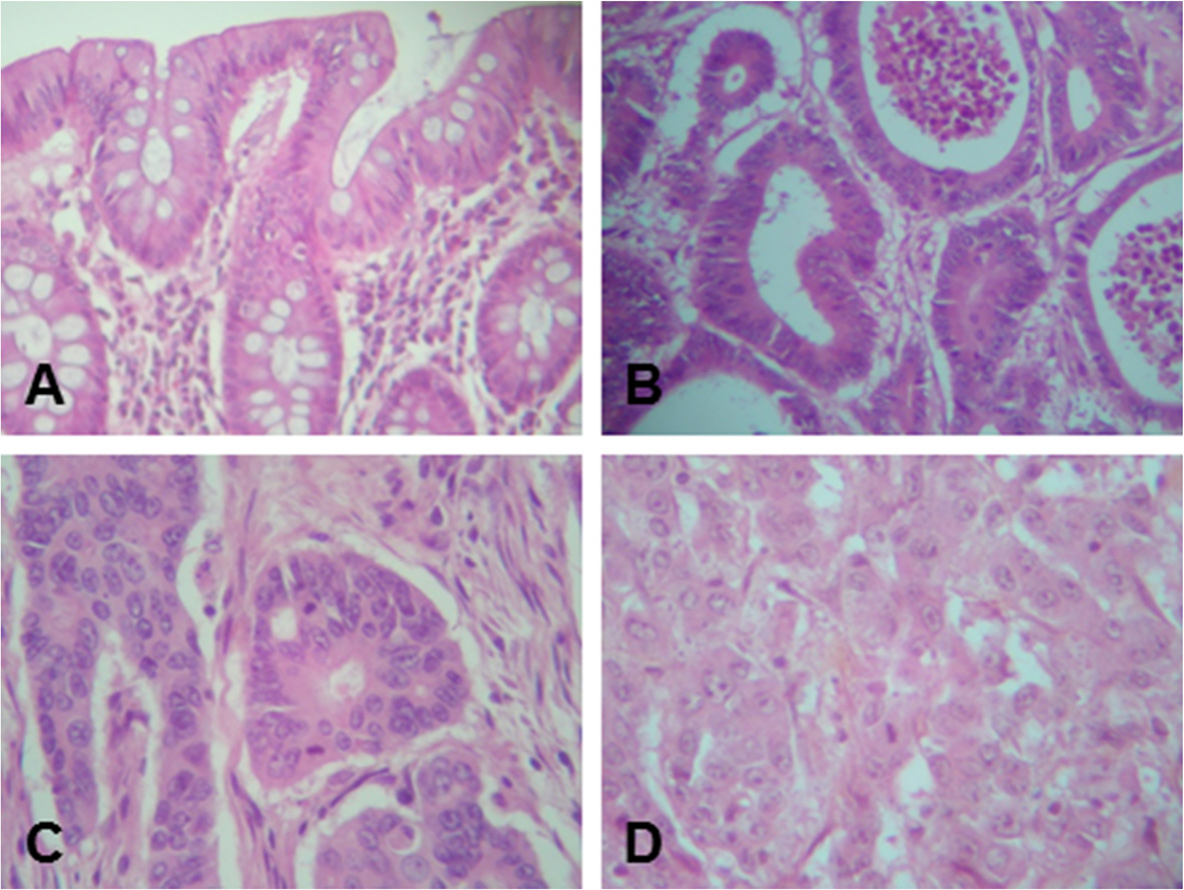
**Histopathological characteristics of colon cancer tissue as compared with normal mucosa.** Representative micrographs of formalin-fixed and paraffin-embedded tissues counterstained with hematoxylin and eosin. **(A)** Normal tissue taken 5-cm away from the tumour area (× 10); **(B)** well differentiated tumour (× 10); **(C)** moderately differentiated tumour (× 20). **D**: poorly differentiated tumour (× 20).

The results are summarized in Table 1. Among the 83 GlpeTH patients included in the study, 51.8% were males and 48.2% were females with a mean age of 69.0 years (ranging from 34 to 99 years old). Most of the patients were more than 50 years-old (84.3%), whereas those who were less than 50-years old represented 15.7%. The sigmoid colon was the most common site of tumour (36.1%), the rectum representing 12.0%. When the tumour site was divided into the proximal and the distal colons, the GlpeTH patients were found to be diagnosed with more distal colon cancers (54.2%) than proximal colon cancers (33.8%) (Table 1). Considering the morphological aspect of the tumour cells and how the latter were organised, and the comparison with the normal adjacent mucosa (Figure 1A), the GlpeTH patients were diagnosed with well (Figure 1B; 41.5%), moderate (Figure 1C; 53.2%) and poorly differentiated CRCs (Figure 1D; 5.3%). TNM stage II (60.2%) and pT3 stage (62.6%) were the most common stages.

**Table 1:** Patient characteristics: comparison GlpeTH and MqueTH

	**GlpeTH**	**MqueTH**	**p value**
	**n = 83 (%)**	**n = 118 (%)**	
**Mean age**	69.0	67.9	0.60^(a)^
**Range**	34 - 99	24 - 96	
**Age**			0.94^(b)^
≤ 50 years	13 (15.7)	18 (15.3)	
> 50 years	70 (84.3)	100 (84.7)	
**Sex**			
Male	43 (51.8)	55 (46.6)	0.47^(b)^
Female	40 (48.2)	63 (53.4)	
**Histological type**			0.073^(c)^
Adenocarcinoma	77 (96.4)	98 (83.0)	
Mucinous adenocarcinoma	5 (2.4)	11 (9.3)	
Undifferentiated carcinoma	1 (1.2)	9 (7.7)	
**Tumour sites**			0.65^(c)^
Right colon	25 (30.1)	34 (28.8)	
Left colon	15 (18.1)	17 (14.4)	
Transverse colon	3 (3.7)	9 (7.7)	
Sigmoid colon	30 (36.1)	39 (33.0)	
Rectum	10 (12.0)	19 (16.1)	
**Tumour grade**			**<**0.0001^(c)^
Well differentiated	32 (41.5)	83 (84.7)	
Moderately differentiated	41 (53.2)	12 (12.2)	
Poorly differentiated	4 (5.3)	3 (3.1)	
**pTNM**			0.045^(c)^
0 /I	5 (6.0)	11 (9.3)	
II	50 (60.2)	57 (48.3)	
III	27 (32.5)	38 (32.2)	
IV	1 (1.2)	12 (10.2)	
**pT stage**			<0.0001^(c)^
T0-T2	12 (14.5)	30 (25.2)	
T3	52 (62.6)	31 (26.5)	
T4	19 (22.9)	57 (48.3)	

Data are given as number and percentage (in brackets) based on the total number of cases in GlpeTH and MqueTH. ^(a)^Student t test. ^(b)^Fisher’s exact test. ^(c)^Chi-square test.

Among the 118 MqueTH patients included in the study (Table 1), 53.4% were females and 46.6% were males with a median age of 67.9 years (ranging from 24 to 96 years old). Interestingly also, 15.3% of the patients were less than 50 years old. Sigmoid colon was also the most common site of tumour (33.0%), rectum representing 16.1%. Approximately, equivalent percentages of distal colon cancers (47.4%) and proximal colon cancers (36.5%) were diagnosed. The latter was often found associated with greater aggressiveness [16]. Regarding the tumour grade, 84.7% of the CRC cases were well differentiated, 12.2% moderately differentiated, and 3.1% poorly differentiated (Figure 1 and Table 1). pT4 stage (48.3%) was the most common stage (Table 1). Thus, the higher percentages of patients diagnosed with mucinous adenocarcinomas, well differentiated CRCs and pTNM stage IV CRCs were seen in the MqueTH as compared with the GlpeTH.

### p53 immunohistochemical analyses

#### Pattern of expression

**Figure 2:**
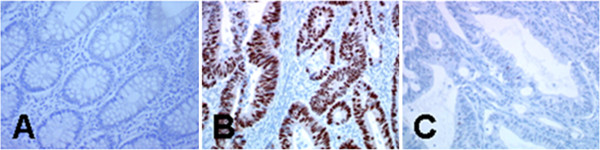
**Immunohistochemical staining pattern of p53. (A)** Normal adjacent tissue (× 20); **(B)** high positive staining detecting in the nucleus of the tumour cells (× 10); **(C)** negative control without the primary antibody (× 20).

**Figure 3:**
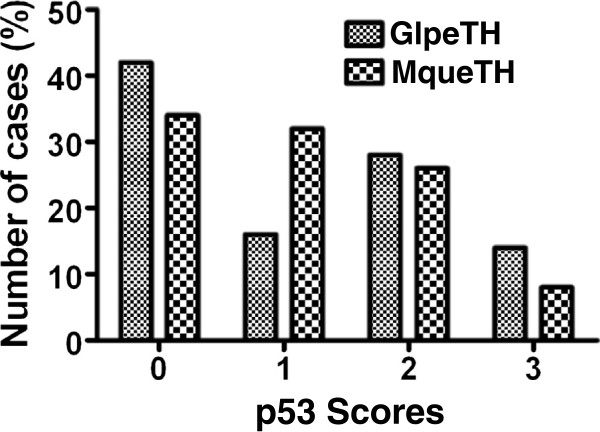
**p53 staining analysis using a scoring method based on nuclear staining intensity.** (0) negative staining, (1) weak staining, (2) moderate staining, (3) strong staining.

Figures 2 and 3 show the results obtained for the p53 staining. We did not find any staining in the adjacent normal mucosa (Figure 2A). p53 staining was specific to tumour cells and was detected exclusively in the nuclei (Figure 2B) compared with the negative control (Figure 2C). Among the 76 CRCs detected in the GlpeTH, 32 cases (42.1%) were p53 negative and 44 cases (57.8) p53 positive. Among the positive cases, 16.0% were weakly stained (+), 28.0% were moderately stained (++) and 14.0% were strongly stained (+++) (Figure 3). In the MqueTH, p53 was negative in 34.2% (40/117) of the CRCs and positive in 65.8% (77/117). Among the positive cases, 32.0% were weakly stained, 26.0% were moderately stained, and 8.0% were strongly stained (Figure 3). Thus, strong nuclear p53 positive tumours (+++) were more frequent in GlpeTH CRCs (14.5%) than in MqueTH CRCs (8.5%) although, this difference did not reach statistical significance (p = 0.069).

#### Association with clinicopathological features

We further analysed the relationship between the p53 nuclear accumulation and the clinicopathological variables studied above. In the case of the GlpeTH patients, there was no significant association between the p53 accumulation and any of the pathological variables, although cancers localised on the distal colon tended to be more frequently p53 positive (61.4%) than cancers localised on the proximal colon (31.8%) (Table 2; p = 0.73). Likewise, CRC diagnosed at early pTNM stage had a greater tendency to be p53 positive (65.9%) than CRC diagnosed at late pTNM stage (34.1%) (Table 2; p = 0.92).

**Table 2:** Association of p53 with tumour site and pTNM in each Teaching Hospital

**Parameters**	**GlpeTH n = 76**			**MqueTH n = 117**		
	**p53 positve**	**p53 negative**	**p**	**P53 positive**	**P53 negative**	**p**
	**n (%)**	**n (%)**		**n (%)**	**n (%)**	
**Tumour site**			0.73^(c)^			0.64^(c)^
Proximal colon	14 (31.8)	13 (40.6)		27 (35.3)	17 (42.5)	
Distal colon	27 (61.4)	17 (53.5)		37 (48.0)	17 (42.5)	
Rectum	3 (6.8)	2 (5.9)		13 (16.9)	6 (15.0)	
**pTNM**			0.92^(c)^			0.029^(c)^
0-I	2 (4.5)	2 (6.2)		4 (5.2)	7 (17.5)	
II	27 (61.4)	20 (62.5)		43 (55.8)	14 (35.0)	
III - IV	15 (34.1)	10 (31.3)		30 (39.0)	19 (47.5)	

Data are given as number and percentage (in brackets) based on the total number of positive cases in GlpeTH and in MqueTH. ^(c)^Chi-square test.

In the case of the MqueTH patients, approximately equivalent percentages of tumour localised on the distal and proximal colons were found to be nuclear p53 positive (Table 2; 42.8% vs 35.3%; p = 0.64). Interestingly however, a significant association between p53 nuclear accumulation and the pTNM stage was observed in the latter patients (p = 0.029), indicating that the proportion of CRC negative for nuclear p53 increased with progression of the CRC stages (Table 2).

Finally, the overall patient population (GlpeTH and MqueTH: 193 patients) was studied (Table 3). No significant association between the p53 expression and any of the pathological variables analysed was found, except for tumour site. Indeed, either in univariate analysis (Table 3; p = 0.026) or multivariate logistic regression analysis (Table 4; OR = 1.89; 95% CI = 1.03-3.46; p = 0.039), the patients whose tumours were diagnosed either on sigmoid colon or rectum were 1.89 times more likely to be positive for p53 nuclear accumulation than if the tumours were diagnosed on colon (left, right and transverse).

**Table 3:** Association of p53 expression with clinicopathological parameters

**Parameters**	**GlpeTH and MqueTH**		
	**p53positve**	**p53negative**	**p value**
	**n = 121 (%)**	**n = 72 (%)**	
**Mean age**	67.2	70.8	0.10^(a)^
**Range**	24 - 99	31 - 93	
**Age**			0.68^(b)^
≤ 50 years	20 (16.5)	10 (13.9)	
> 50 years	101 (83.5)	62 (86.1)	
**Sex**			1.0^(b)^
Male	60 (49.6)	35 (48.6)	
Female	61 (50.4)	37 (51.4)	
**Histologycal type**			0.38^(c)^
ADK	110 (90.9)	61 (84.7)	
Mucinous ADK	8 (6.6)	7 (9.7)	
Other	3 (2.5)	4 (5.6)	
**Tumour site**			0.072^(c)^
Colon*	55 (45.5)	45 (62.5)	
sigmoid	46 (38.0)	19 (26.4)	
Rectum	20 (16.5)	8 (11.1)	
**Tumour site**			0.026^(b)^
Colon* yes	55 (45.5)	45 (62.5)	
Colon no	66 (54.5)	27 (37.5)	
**pTNM**			0.11^(c)^
0-I	6 (5.0)	9 (12.5)	
II	70 (57.8)	34 (47.2)	
III - IV	45 (37.2)	29 (40.3)	
**Teaching Hospital**			0.29^(b)^
Guadeloupe	44 (37.7)	32 (43.1)	
Martinique	77 (62.3)	40 (56.9)	

ADK: adenocarcinoma. Data are given as number and percentage (round brackets) based on the total number of p53 positive and p53 negative cases in GlpeTH and MqueTH. ^(a)^Student t test. ^(b)^Fisher’s exact test. ^(c)^Chi-square test. *right, left and transverse.

**Table 4:** Multivariate logistic regression analysis

	**OR**	**CI 95%**	**P value**
**Age**	0.987	0.967 - 1.007	0.20
**Sex**			0.80
Female	1.079	0.592 - 1.976	
Male			
**Tumour site**			0.039
Colon* yes	1.891	1.033 - 3.463	
Colon no			
**pTNM**			0.38
0,I,II or III	1.831	0.471 - 7.118	
IV			
**Teaching Hospital**			0.37
Guadeloupe	1.324	0.715 - 2.449	
Martinique			

*right, left and transverse.

## Discussion

Our data indicate disparities between the GlpeTH and the MqueTH patients regarding CRC cartography. To our knowledge, no other study has investigated and compared the clinicopathological features and the p53 pattern of expression of CRC in these populations.

There were no differences in age (p = 0.94), sex (p = 0.47), histology (p = 0.073) and tumour sites (p = 0.65) between CRCs in the GlpeTH and the MqueTH patients, although the percentage of men was slightly higher in the former patients (51.8%) than in the latter patients (46.6%) and, inversely the percentage of women was slightly lower (48.2% vs 53.4%). Approximately equivalent percentages of proximal colon (36.5%) and distal colon cancers (47.4%) were diagnosed in the MqueTH patients as compared with the high proportion of distal colon cancers diagnosed in the GlpeTH patients (54.2%). In accordance with this latter result, Dieye et al. [11] report that, among the 138 Guadeloupian patients recruited in 2008 (cancer registry data), 58.9% were males and 41.1% females, and that distal colon cancers were more frequent. Also, the data of Dieye et al. [11,14] and Ngasseu et al. [13] confirm our results obtained with the MqueTH patients.

Our non-standardized results were also compared with those obtained for patients in Metropolitan France [11,17,18] and for Caucasian patients [1,2,4]. The proportion of colon cancer cases occurring before the age of 50 was found to be higher in the French departments (15% vs 2-6%), whereas the proportion of rectal cancer was found to be lower (12–16.1% vs 23-27%). Furthermore, compared with the Caucasian patients, African Americans are typically diagnosed with CRC at a younger age, with a higher incidence of tumours located on the proximal colon and with well-differentiated CRC [19-23]. Tanzanian patients (African) are diagnosed at a young age as well, however, moderately differentiated and rectosigmoid tumours in those patients were mostly represented [24].

Histopathological data was further discussed. Significant differences were found between the MqueTH and the GlpeTH patients regarding tumour grade (p < 0.0001), considered as an independent prognostic factor [4], tumour stage (p = 0.045) and depth of tumour invasion (p < 0.0001). Moderately (53.2%) and poorly differentiated CRCs (5.3%) were diagnosed in the GlpeTH patients, whereas CRCs were at early pTNM stages (66.2%). By contrast, well differentiated CRCs (84.7%) were more frequently diagnosed in the MqueTH patients, whereas pTNM stage IV CRCs were highly represented (10.2%). As a reminder, the value obtained for African American and Caucasian patients was 14% and for Metropolitan France patients it was 13-22% [17-22].

As far as p53, the “guardian of the genome”, is concerned [25], reviews of the literature indicate that its clinical significance in CRC is still raising a controversial debate, probably due to the mode of patient selection, the origin of patients, tumour sites or the use of different p53 antibodies for IHC [6,26-32]. For example, the study of Iacopetta [6] indicates that a high frequency of p53 mutation was observed in distal colon and rectal cancer; however, these alterations in the p53 gene are likely to have very little or no prognostic significance in CRC patients treated with surgery alone. This could be the case for the GlpeTH and the MqueTH patients included in the present study. On the other hand, the study of Mane et al. [31] indicates that approximately equivalent proportions of distal and proximal colon cancers were positive for p53 in African American patients, whereas distal colon cancer from white patients were more frequently positive for p53 than proximal colon cancer was. The authors conclude that nuclear p53 was a valuable indicator of poor prognosis only for white patients with tumours located on the proximal colon. Diez et al. [32] show that p53 overexpression was more frequent in distal than in proximal tumours, and the authors concluded that p53 exhibited different prognostic values in distal and proximal colon. Nevertheless, when we used the monoclonal DO-1 p53 antibody to visualize the p53 expression and location, our IHC analyses indicated that p53 overexpression was observed in 57.9% and 65.8% of the CRCs of the GlpeTH and the MqueTH patients, respectively, which is in accordance with the 40-81% range of p53 positivity observed in previous reports [9,10,27-32]. Staining was restricted to the nuclei of malignant cells. Regarding the GlpeTH patients, 61.4% of the distal colon cancers were p53 positive, versus 31.8% of the proximal colon cancers. Regarding the MqueTH patients, equivalent proportions of the distal and the proximal colon cancers were positive for p53. The paradox noticed for the clinical, pathological data was also found for the p53 staining pattern. Indeed, the MqueTH data were, in part, close to those observed for African Americans, although the Martinican population is highly mixed [15].

## Conclusions

This retrospective, descriptive, comparative study has enabled us to map the clinicopathological characteristics of CRC in two groups of patients. The GlpeTH patients were diagnosed with more moderated CRCs, a high percentage of distal colon cancers, among which 61.4% were p53 positive, but with few pTNM IV stages. By contrast, the MqueTH patients were diagnosed with more differentiated tumours, equivalent percentages of proximal and distal colon cancers which were found equally positive for p53, but with many pTNM IV stages. This paradox may be due to differences in tumour location (distal vs proximal), a multiplicity of genetic profiles of patients, or patients who were not treated locally. Even if our study is limited due to its small size, it emphasizes the originality of our results and should alert the physicians of the GlpeTH and the MqueTH regarding patient management.

## Abbreviations

CRC: Colorectal cancer; GlpeTH: Guadeloupe teaching hospital; MqueTH: Martinique teaching hospital; DMI: Department of medical information; IHC: Immunohistochemistry; DAB: Diaminobenzidine; OR: Odds ratio; CI: Confidence interval.

## Competing interests

The authors declare having no competing interests.

## Authors’ contributions

MD supervised and participated in the study design, result interpretation and wrote the manuscript. MO contributed to the clinical data and the immunohistochemical analysis. AMA carried out the immunohistochemical experiments. BT performed the statistical analysis. JBV, JD and ML contributed to the clinical and demographic data base. JSR participated in the study design, manuscript editing. All authors read and approved the final manuscript.

## Pre-publication history

The pre-publication history for this paper can be accessed here:

http://www.biomedcentral.com/1472-6890/14/12/prepub
